# Prognostic Value and Clinicopathological Features of MicroRNA-206 in Various Cancers: A Meta-Analysis

**DOI:** 10.1155/2020/2159704

**Published:** 2020-10-20

**Authors:** Rongqiang Liu, Shiyang Zheng, Shengjia Peng, Yajie Yu, Jianwen Fang, Siwen Tan, Fan Yao, Zhihua Guo, Yi Shao

**Affiliations:** ^1^Department of Ophthalmology, The First Affiliated Hospital of Nanchang University, Nanchang, 330006 Jiangxi, China; ^2^Department of Hepatobiliary Surgery, The First Affiliated Hospital of Guangzhou Medical University, Guangzhou, 510220 Guangdong, China; ^3^Department of Breast Surgery, The Third Affiliated Hospital of Guangzhou Medical University, Guangzhou 510150, China; ^4^Department of Thoracic Oncology, The First Affiliated Hospital of Guangzhou Medical University, Guangzhou 510220, China

## Abstract

It has been reported that microRNA-206(miR-206) plays an important role in cancers and could be used as a prognostic biomarker. However, the results are controversial. Therefore, we summarize all available evidence and present a meta-analysis to estimate the prognostic value of miR-206 in various cancers. The relevant studies were collected by searching PubMed, EMBASE, and Web of Science databases until August 21, 2020. Hazard ratios (HRs) and odds ratios (ORs) with 95% confidence intervals (CIs) were applied to explore the association between miR-206 and survival results and clinicopathologic features. Sources of heterogeneity were investigated by subgroup analysis and sensitivity analysis. Publication bias was evaluated using Egger's test. Twenty articles involving 2095 patients were included in the meta-analysis. The pooled HR showed that low miR-206 expression was significantly associated with unfavourable overall survival (OS) (HR = 2.03, 95 CI%: 1.53-2.70, *P* < 0.01). In addition, we found that low miR-206 expression predicted significantly negative association with tumor stage (III-IV VS. I-II) (OR = 4.20, 95% CI: 2.17-8.13, *P* < 0.01), lymph node status (yes VS. no) (OR = 3.58, 95%: 1.51-8.44, *P* = 0.004), distant metastasis (yes VS. no) (OR = 3.19, 95%: 1.07-9.50, *P* = 0.038), and invasion depth (T3 + T4 vs. T2 + T1) (OR = 2.43, 95%: 1.70-3.49, *P* < 0.01). miR-206 can be used as an effective prognostic indicator in various cancers. Further investigations are warranted to validate the present results.

## 1. Introduction

MicroRNAs (miRNAs) are a class of small noncoding single-stranded RNAs (20 to 24 nucleotides) with the function of regulating gene expression by binding to the 3′-UTR of the target mRNA [1]. miRNA plays an indispensable role in differentiation, proliferation, metabolism, hemostasis, apoptosis, and inflammation [2–6]. Increasing evidence has shown that miRNAs play an important role in tumor progression and can be used for clinical purposes such as diagnosis and prognosis of tumors [7–9]. Among them, miR-206 is one of the most attractive miRNAs.

miR-206 is a 21-nucleotide miRNA molecule, located on the human chromosome 6p12. 2 [10]. miR-206 was first discovered in skeletal muscle and belonged to one of the members of the “muscle-specific miRNA (myomiR)” family [11]. miR-206 is considered to be a tumor suppressor and downregulated in a variety of tumors. Fact has disclosed that miR-206 participates in tumor cell proliferation, differentiation, invasion, metastasis, and other processes by regulating genes related to cell cycle, division, and apoptosis, such as Cyclin D2, MET, STAT3, and VEGF [12]. Additionally, more and more studies have found that low miR-206 expression was significantly associated with unfavourable prognosis in cancers, such as malignant astrocytomas, melanoma, gastric cancer (GC), colorectal cancer (CRC), osteosarcoma, acute myeloid leukemia (AML), cervical cancer (CC), nonsmall cell lung cancer (NSCLC), renal clear cell carcinoma (RCC), and esophageal squamous cell carcinoma (ESCC) [13–28]. However, several other studies have reached the opposite conclusion [29–32]. At present, the prognostic values of miR-206 in cancers have still not been fully elucidated. In this study, we conducted a meta-analysis to synthetically evaluate the clinicopathological and prognostic values of miR-206 in cancers.

## 2. Material and Methods

### 2.1. Search Strategy

Articles in electronic databases (PubMed, EMBASE, and Web of Science) published until August 21, 2020, were searched using the following keywords: “MicroRNA-206 OR miR-206” OR “miRNA-206” AND “cancer OR carcinoma OR neoplasm OR tumor OR tumor”. Language restrictions were set in English. The titles, abstracts, full texts, and the possible reference lists were screened to identify qualified studies. The study was implemented according to the Preferred Reporting Items for Systematic Reviews and Meta-Analyses (PRISMA) guidelines.

### 2.2. Inclusion and Exclusion Criteria

Three researchers (RQ.L, SHY.ZH, and SHJ.P) independently conducted the literature search, and disagreements were resolved by consensus. The inclusion criteria were as follows: (1) they investigated the relationship between miR-206 with survival outcome in any type of cancer; (2) they categorized patients into low and high-expression groups based on the miR-206 expression; (3) they provided sufficient data to calculate the hazard ratio (HR) and the 95% confidence interval (CI); (4) they detected the expression of miR-206 in human tumor tissue or serum; and (5) they were published in English. The exclusion criteria were as follows: (1) they provided insufficient data to calculate HR and the 95% CI; (2) they were case abstract, case reports, conference papers, reviews, letters, published in non-English languages, and data from the public databases; (3) they were duplicated or overlapped studies; and (4) they were laboratory studies on cell lines or animals level.

### 2.3. Data Extraction and Quality Assessment

Three researchers (RQ.L, SHY.ZH, and SHJ.P) independently checked the included studies and extracted the required data. The relevant information included the name of the first author, publication year, country, study design, tumor type, sample size, detected sample, analysis type, detection method, overall survival (OS), disease-free survival/progression-free survival (DFS/PFS)), hazard ratio (HR), odds ratios (OR), and the corresponding 95% CI. For studies reporting the results of both univariate and multivariate analyses, the multivariate analysis was selected as it was more accurate. We assessed the quality of each study according to the Newcastle–Ottawa Quality Assessment Scale (NOS) [33]. NOS scores of 0–3, 4–6, and 7–9 denoted low, moderate, and high quality, respectively.

### 2.4. Statistical Analysis

All data analyses were performed using the STATA version 12.0 software (Stata Corporation, College Station, TX, USA). HR, OR, and their corresponding 95% CI were used to analyze the pooled data. Statistical variables described in the study were used directly in our analysis. Otherwise, we used the Engauge Digitizer version 4.1 to extract data from graphical survival plots according to the methods described by Tierney et al. [34]. A forest plot was used to explore the prognostic role of miR-206 in cancers. A fixed-effects model was used when *I*^2^ was <50%. Otherwise, a random-effects model was adopted. Subgroup analyses were performed to explore the sources of heterogeneity. Sensitivity analysis was used to verify the stability of the meta-analysis. The funnel plot and Egger's test were used to assess publication bias. *P* < 0.05 denoted statistical significance.

## 3. Results

### 3.1. Literature Search

**Figure 1:**
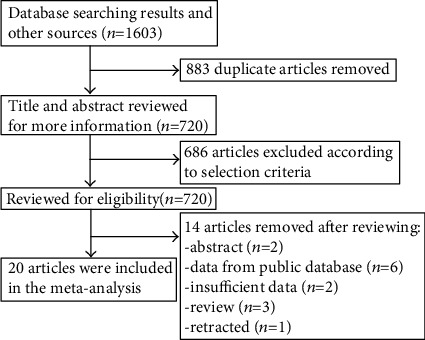
Flow diagram of the literature search.

Through a systematic literature search of designated databases, we primarily identified a total of 1603 articles. After the removal of 883 duplicate publications, 720 articles remained. We further excluded 686 articles by browsing the titles and abstracts. After full-text review, fourteen articles were further excluded. Finally, twenty retrospective articles published from 2010 to 2020 were included in the meta-analysis. The flow diagram of the literature search is shown in Figure 1.

### 3.2. Study Characteristics

The total number of patients in the included studies was 2089 (range: 41–372 patients). Eighteen studies were produced in China, and two in Europe. Thirteen studies detected the expression of miR-206 in tumor tissues, and seven studies in serum. All articles used polymerase chain reaction (PCR) to detect the miR-206 expression. The pooled HR of eleven studies adopted multivariate analysis, and nine used univariate analysis. Ten studies directly provided the HR and 95% CI. These had to be extracted from the survival curve in the remaining eight articles. Twelve different cancers were assessed in this study, including rhabdomyosarcomas (RMS) [32], malignant astrocytomas [13], melanoma [14], GC [15, 18, 22], CRC [16, 19], osteosarcoma [17], RCC [26, 27, 29], AML [24], CC [20, 21, 23, 30], breast cancer (BC) [31], NSCLC [25], and ESCC [28]. The mean NOS scores of the included studies were 6.5. The basic study data are shown in Table 1.

### 3.3. Meta-Analysis Findings

#### 3.3.1. Low miR-206 Expression and OS

**Figure 2:**
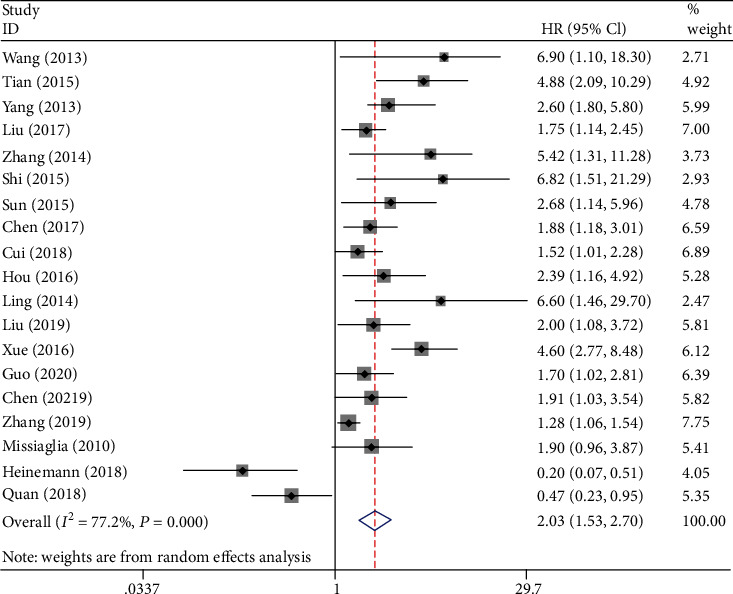
Forest plot of the relationship between low miR-206 expression and OS.

Nineteen studies involving 1964 patients explored the relationship between miR-206 expression and prognosis using OS. We used a random-effects model to calculate the pooled HR (95% CI) owing to moderate heterogeneity (*I*^2^ = 77.2%). The results of the meta-analysis revealed that low miR-206 expression was significantly associated with unfavourable OS (HR = 2.03, 95 CI%: 1.53-2.70, *P* < 0.01). The forest plot is shown in Figure 2.

#### 3.3.2. Subgroup Analysis for OS

We conducted subgroup analysis based on cancer type, analysis type, race, detected sample, source of HR, and sample size. The results were shown in Table 2. The findings revealed that low miR-206 expression indicated poorer OS in the subgroups of GC (HR = 2.79, 95% CI:1.82-4.30) (Supplemental Figure 1), CRC (HR = 1.89, 95% CI: 1.33-2.67) (Supplemental Figure 2), CC (HR = 1.76, 95% CI: 1.30-2.38)(Supplemental Figure 3), multivariate analysis (HR = 2.24,95% CI: 1.85-2.72)(Supplemental Figure 4), Asian (HR = 2.23,95% CI: 1.69-2.93) (Supplemental Figure 5), tissue (HR = 2.05, 95% CI: 1.49-2.82) (Supplemental Figure 6), data from reported (HR = 2.92, 95% CI: 2.10-4.06) (Supplemental Figure 7), sample size ≥ 100 (HR = 2.82, 95% CI: 1.34-5.90), and sample size < 100 (HR = 1.79, 95% CI: 1.35-2.38) (Supplemental Figure 8). As for the other subgroups, we did not observe any statistical differences. In addition, we noted the absence of heterogeneity in stratified studies with GC and CRC (*I*^2^ = 0 and 0, respectively). Therefore, we believe that cancer type may be the source of heterogeneity.

#### 3.3.3. Low MicroRNA-206 Expression and DFS/PFS

**Figure 3:**
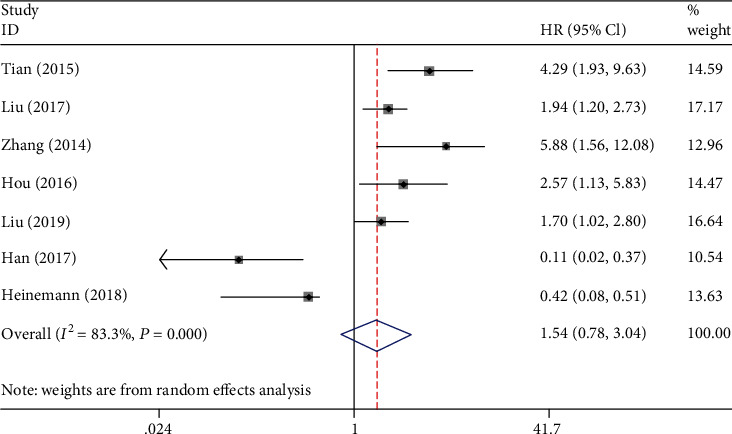
Forest plot of the relationship between low miR-206 expression and DFS/PFS.

Seven studies involving 698 patients documented the relationship between miR-206 expression and prognosis using DFS/PFS. We used a random-effects model to calculate the pooled HR (95% CI) owing to the obvious heterogeneity (*I*^2^ = 83.3%). The results showed that low miR-206 expression did not exhibit a significant association with DFS/PFS (HR: 1.54, 95% CI: 0.78–3.04, *P* = 0.216). The forest plot is illustrated in Figure 3.

#### 3.3.4. Low MicroRNA-206 Expression and Clinicopathological Features

We summarized data regarding the association between low miR-206 expression and clinicopathological features, including gender, age, tumor diameter, tumor stage, tumor differentiation, lymph node status, distant metastasis, and invasion depth metastasis. The results were displayed in Table 3. The pooled OR showed that low miR-206 expression had a negative association with tumor stage (III-IV VS. I-II) (OR = 4.20, 95% CI: 2.17-8.13, *P* < 0.01), lymph node status (yes VS. no) (OR = 3.58, 95%: 1.51-8.44, *P* = 0.004), distant metastasis (yes VS. no) (OR = 3.19, 95%: 1.07-9.50, *P* = 0.038), and invasion depth (T3 + T4 vs. T2 + T1) (OR = 2.43, 95%: 1.70-3.49, *P* < 0.01). Furthermore, we also observed there was no significant association between low miR-206 expression and gender (male VS. female) (OR = 0.90, 95 CI%: 0.68-1.17, *P* = 0.421), age (old VS. young) (OR = 1.23, 95% CI: 0.96-1.59, *P* = 0.101), tumor diameter (big vs. small) (OR = 1.39, 95% CI: 0.83-2.32, *P* = 0.215), and tumor differentiation (poor VS. moderate/well) (OR = 1.30, 95% CI: 0.71-2.38, *P* = 0.398).

### 3.4. Sensitivity Analysis

**Figure 4:**
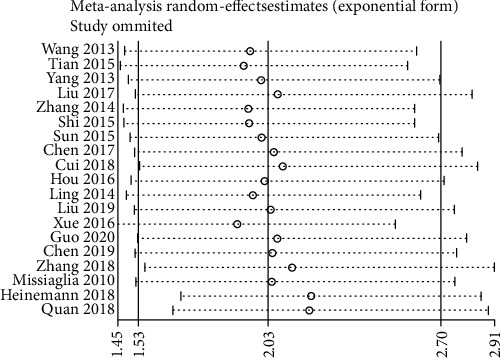
Sensitivity analysis for OS.

**Figure 5:**
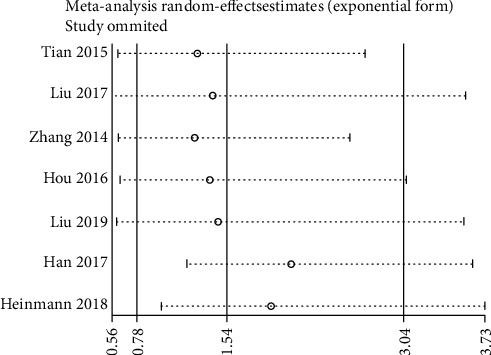
Sensitivity analysis for DFS/PFS.

We implemented sensitivity analysis by sequentially deleting each of the included studies. The results for OS were consistent with the comprehensive analysis, confirming that our results were stable (Figure 4). However, sensitivity analysis for DFS/PFS showed that the results were unstable (Figure 5).

### 3.5. Publication Bias

**Figure 6:**
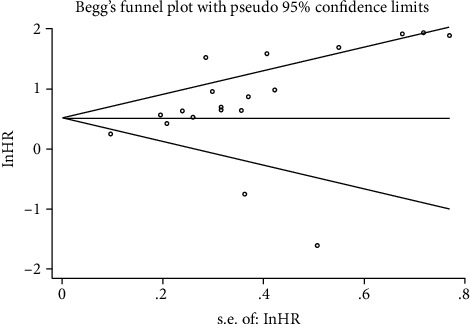
Funnel plots for publication bias for OS.

**Figure 7:**
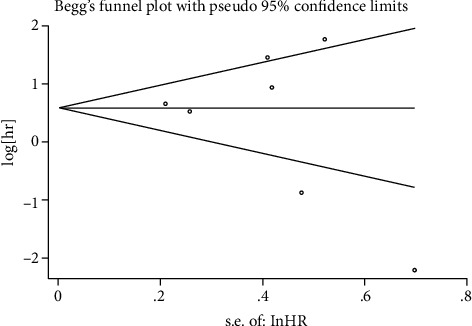
Funnel plots for publication bias for DFS/PFS.

The funnel plots were used to qualitatively assess the publication bias for OS or DFS/PFS, and Egger's test was applied to quantify the publication bias. The *P* value of Egger's test was 0.051 for OS (Figure 6) and 0.520 for DFS/PFS (Figure 7). *P* was more than 0.05, and no significant bias was observed.

## 4. Discussion

Cancer has surpassed all other diseases and has become the leading cause of death worldwide. According to the survey, there were 18.1 million new cancer cases and 9.6 million cancer deaths worldwide in 2018 and showed a clear upward trend in developing countries [35]. It is urgent to find effective ways of prevention and treatment. Studies have confirmed that miRNA-206 plays a very important role in the development of tumors. miR-206 is involved in cell proliferation, differentiation, and metastasis by inhibiting mRNA translation or directly degrading mRNA through incompletely pairing with the 3′-untranslated region of the targeted mRNA [36]. Our meta-analysis indicated that miR-206 can effectively predict the prognosis of different tumors. Prognostic markers are helpful for the early identification of high- and low-risk patients, resulting in individualized treatment for each patient. As a novel prognostic marker, we believe miR-206 may assist physicians in comprehensively evaluating patients' condition and more accurately predicting clinical outcomes and may serve as a new therapeutic target.

To the best of our knowledge, our study is the first meta-analysis to explore the prognostic value of miR-206 in various tumors. The comprehensive analysis found that low miR-206 expression was significantly associated with unfavourable OS (HR = 2.20, 95 CI%: 1.53-3.16, *P* < 0.01). Subgroup analysis for OS showed that low miR-206 expression mainly displayed the adverse prognosis in GC (HR = 2.79, 95% CI: 1.82-4.30), CRC (HR = 1.89, 95% CI: 1.33-2.67), and CC (HR = 1.76,95% CI: 1.30-2.38), indicating that miR-206 has better predictive effect for the three types of tumors. In order to exclude the influence of different races, we separately analyzed the yellow and the white race. The results showed that the low miR-206 expression was closely associated with poor prognosis in the yellow race (HR = 2.23, 95% CI: 1.69-2.93), but not in the white race (HR = 0.635, 95% CI: 0.07-5.758), suggesting that the results were more applicable to the yellow race based on existing evidence. In addition, we found that low miR-206 expression exhibited no significant association with DFS/PFS. However, the sensitivity analysis for DFS/PFS indicated that the results were not stable. We speculate that it may be related to the limited studies and the quality of the researches. However, both sensitivity analysis and publication bias for OS proved that the comprehensive results were very stable. In view of the above results, we have sufficient reasons to believe that miR-206 is a suitable and effective prognostic indicator of cancers for clinical application.

We also summarized the relationship between low miR-206 expression and clinical features. Studies have shown that low miR-206 expression presented obvious association with tumor stage (III-IV VS. I-II), lymph node status (yes VS. no), distant metastasis (yes VS. no), and invasion depth (T3 + T4 vs. T2 + T1). We thought that miR-206 might affect tumor progression by participating in tumor differentiation, invasion, and metastasis.

Several studies have explored the specific mechanisms of miR-206 in tumors. Ren et al. found that miR-206 can inhibit the proliferation, invasion, and metastasis of CRC cells by targeting FMNL2 and c-MET [37]. Liang et al. reported that miR-206 inhibited triple-negative breast cancer cell invasion and angiogenesis through downregulating vascular endothelial growth factor (VEGF), mitogen-activated protein kinase 3(MAPK3), and SOX9 expression levels [38]. Yang et al. demonstrated that miR-206 downregulated protein tyrosine phosphatase 1B (PTP1B) to inhibit cell proliferation, invasion, and migration in hepatocellular carcinoma [39]. In addition, miR-206 can also restrain the growth of hepatocellular carcinoma by targeting cyclin-dependent kinase 9 (CDK9) [40]. Chen et al. revealed that high miR-2016 expression can weaken the proliferation of drug-resistant gastric cancer cells, facilitate cell apoptosis, and decrease cisplatin resistance via targeted ERK/MAPK signaling pathway [41]. The researchers discovered that miR-206 can also inhibit GC proliferation in part by repressing cyclin D2 (CCND2). Wang and Tian demonstrated that miR-206 suppressed cell proliferation, migration, and invasion by targeting athanogene 3 (BAG3) in CC [42]. The C-Met/AKT/mTOR signaling pathway was confirmed to be one of the mir-206 targeted pathways in epithelial ovarian cancer [43]. The above results show that miR-206 regulates tumor progression through a variety of different signaling pathways and targets, which reflects the complexity of its mechanism.

There were certain limitations in the meta-analysis. Firstly, twenty included studies had small sample sizes, and their results may not be reliable. Secondly, ten studies of the HR and CI values extracted from the survival curve may not be equal to the true value. Thirdly, all included studies were retrospective studies. Fourthly, most studies included in the meta-analysis were conducted in Asia. Future studies involving patients of different races and from various regions are warranted. Finally, sensitivity analysis for DFS/PFS showed that the results were unstable.

This meta-analysis also has some strengths. Firstly, this was the first meta-analysis to investigate the relationship between miR-206 and survival outcomes in cancers. Secondly, sensitivity analysis and publication bias for OS displayed that the results were stable. In addition, our statistical analysis was rigorous and detailed.

In summary, we demonstrated that miR-206 can be used as an effective prognostic indicator in various cancers, especially for GC, CRC, and CC mir-206 may have great application value in clinical tumor prevention, prognosis, and targeted therapy. Undoubtedly, further large-scale, prospective, multicentric, and well-designed studies are warranted to validate the results.

## Figures and Tables

**Table 1:** Basic information of eligible studies for miR-206.

Study	Year	Country	Study type	Tumor type	Sample size	Detected sample	Detected method	Analysis type	Survival analysis	Source of HR	NOS score
Wang	2013	China	R	Astrocytomas	108	Tissue	qRT-PCR	Univariate	OS	Reported	6
Tian	2015	China	R	Melanoma	60	Serum	qRT-PCR	Multivariate	OS, DFS	Reported	6
Yang	2013	China	R	GC	98	Tissue	qRT-PCR	Multivariate	OS	Reported	7
Liu	2017	China	R	CRC	73	Serum	qRT-PCR	Multivariate	OS, DFS	Reported	6
Zhang	2014	China	R	Osteosarcoma	100	Serum	qRT-PCR	Multivariate	OS, DFS	Reported	6
Shi	2015	China	R	GC	220	Tissue	qRT-PCR	Multivariate	OS	Reported	7
Sun	2015	China	R	CRC	80	Tissue	qRT-PCR	Multivariate	OS	Reported	7
Chen	2017	China	R	CC	41	Tissue	qRT-PCR	Multivariate	OS	SC	7
Cui	2018	China	R	CC	56	Tissue	qRT-PCR	Univariate	OS	SC	6
Hou	2016	China	R	GC	150	Serum	qRT-PCR	Multivariate	OS, DFS	SC	7
Ling	2014	China	R	CC	66	Tissue	qRT-PCR	Multivariate	OS	SC	7
Liu	2019	China	R	AML	73	Serum	qRT-PCR	Univariate	OS, DFS	SC	7
Xue	2016	China	R	NSCLC	116	Tissue	qRT-PCR	Univariate	OS	Reported	6
Guo	2020	China	R	RCC	60	Tissue	qRT-PCR	Multivariate	OS	Reported	7
Chen	2019	China	R	RCC	46	Tissue	qRT-PCR	Univariate	OS	SC	6
Zhang	2019	China	R	ESCC	52	Tissue	qRT-PCR	Univariate	OS	SC	6
Missiaglia	2010	UK	R	RMS	119	Tissue	qRT-PCR	Multivariate	OS	Reported	7
Heinemann	2018	Germany	R	RCC	68	Serum	qRT-PCR	Univariate	OS, PFS	SC	6
Quan	2018	China	R	BC	372	Tissue	qRT-PCR	Univariate	OS	SC	6
Han	2017	China	R	CC	131	Serum	qRT-PCR	Univariate	DFS	SC	7

Abbreviation: R: retrospective; P: prospective; RMS: rhabdomyosarcomas; BC: breast cancer; GC: gastric cancer; RCC: renal cell carcinomas; CRC: colorectal cancer; AML: acute myeloid leukemia; CC: cervical cancer; ESCC: esophageal squamous cell carcinoma; OS: overall survival; DFS: disease-free survival; PFS: progression-free survival; SC: survival curve.

**Table 2:** Subgroup analysis for OS in patients with low miR-206 expression.

Stratified analysis	No. of studies	No. of patients	*P* value	Heterogeneity		
				*I* ^2^ (%)	*P* value	Model
Cancer type						
GC	3	468	≤0.001	0	0.371	Fixed
CRC	2	153	≤0.001	0	0.359	Fixed
CC	3	163	≤0.001	43.5	0.17	Fixed
RCC	3	174	0.912	87.6	≤0.001	Random
Others	8	1000	0.003	85.4	≤0.001	Random
Analysis type						
Univariate analysis	8	891	0.149	85.9	≤0.001	Random
Multivariate analysis	11	1067	≤0.001	33	0.135	Fixed
Race						
Caucasian	2	187	0.686	92.4	≤0.001	Random
Asian	17	1771	≤0.001	73.8	≤0.001	Random
Sample						
Tissue	13	1434	≤0.001	74.8	≤0.001	Random
Serum	6	524	0.068	83	≤0.001	Random
Source of HR						
Reported	10	1034	≤0.001	54.3	0.02	Random
SC	9	924	0.105	77	≤0.001	Random
Sample size						
≥100	7	1185	0.006	81.6	≤0.001	Random
<100	12	773	≤0.001	71.7	≤0.001	Random

**Table 3:** Association between low miR-206 expression and clinicopathological features.

Clinicopathologic features	No. of studies	No. of patients	Estimate OR (95% CI)	*P* value	Heterogeneity		
					*I* ^2^ (%)	*P* value	Model
Gender (male vs. female)	11	1060	0.88 (0.68-1.14)	0.321	0	0.959	Fixed
Age (old vs. young)	11	1028	1.20 (0.94-1.53)	0.137	0	0.495	Fixed
Tumor diameter (big vs. small)	8	634	1.39 (0.83-2.32)	0.215	57.2	0.022	Random
Tumor stage (III-IV vs. I-II)	10	896	4.20 (2.17-8.13)	≤0.001	75	≤0.001	Random
Tumor differentiation (poor vs. moderate/well)	9	798	1.34 (0.77-2.30)	0.299	65.6	0.003	Random
Lymph node status (yes vs. no)	9	728	3.58 (1.51-8.44)	0.004	81.9	≤0.001	Random
Distant metastasis (yes vs. no)	5	516	3.19 (1.07-9.50)	0.038	67	0.016	Random
Invasion depth (T3 + T4 vs. T2 + T1)	4	538	2.43 (1.70-3.49)	≤0.001	0	0.412	Fixed

## Data Availability

All relevant data are within the paper and its Supporting Information files.

## Supplementary Materials

Supplementary 1 Figure S1. Forest plot of the relationship between low miR-206 expression and GC.

Supplementary 2 Figure S2. Forest plot of the relationship between low miR-206 expression and CRC.

Supplementary 3 Figure S3. Forest plot of the relationship between low miR-206 expression and CC.

Supplementary 4 Figure S4. Forest plot of subgroup analysis based on multivariate analysis.

Supplementary 5 Figure S5. Forest plot of subgroup analysis based on Asian.

Supplementary 6 Figure S6. Forest plot of subgroup analysis based on tissue.

Supplementary 7 Figure S7. Forest plot of subgroup analysis based on data from reported.

Supplementary 8 Figure S8. Forest plot of subgroup analysis based on sample size.

## References

[1] Bartel D. P. (2004). MicroRNAs: genomics, biogenesis, mechanism, and function. *Cell*.

[2] Novák J., Olejníčková V., Tkáčová N., Santulli G. (2015). Mechanistic role of microRNAs in coupling lipid metabolism and atherosclerosis. *Advances in Experimental Medicine and Biology*.

[3] Tétreault N., de Guire V. (2013). miRNAs: their discovery, biogenesis and mechanism of action. *Clinical Biochemistry*.

[4] Chen Y., Fu L. L., Wen X. (2014). Oncogenic and tumor suppressive roles of microRNAs in apoptosis and autophagy. *Apoptosis*.

[5] Weiner A. M. J. (2018). MicroRNAs and the neural crest: from induction to differentiation. *Mechanisms of Development*.

[6] Teruel-Montoya R., Rosendaal F. R., Martínez C. (2015). MicroRNAs in hemostasis. *Journal of Thrombosis and Haemostasis*.

[7] Wang W., Li J., Zhu W. (2014). MicroRNA-21 and the clinical outcomes of various carcinomas: a systematic review and meta-analysis. *BMC Cancer*.

[8] Shao Y., Gu W., Ning Z., Song X., Pei H., Jiang J. (2017). Evaluating the prognostic value of microRNA-203 in solid tumors based on a meta-analysis and the Cancer Genome Atlas (TCGA) Datasets. *Cellular Physiology and Biochemistry*.

[9] Ma X., Bai J., Xie G., Liu Y., Shuai X., Tao K. (2017). Prognostic significance of microRNA-101 in solid tumor: a meta-analysis. *PLoS One*.

[10] Pan J. Y., Sun C. C., Bi Z. Y. (2017). miR-206/133b cluster: a weapon against lung cancer?. *Molecular Therapy Nucleic Acids*.

[11] Ma G., Wang Y., Li Y. (2015). miR-206, a key modulator of skeletal muscle development and disease. *International Journal of Biological Sciences*.

[12] Tang R., Ma F., Li W., Ouyang S., Liu Z., Wu J. (2017). miR-206-3p inhibits 3T3-L1 cell adipogenesis via the c-Met/PI3K/Akt pathway. *International Journal of Molecular Sciences*.

[13] Wang S., Lu S., Geng S., Ma S., Liang Z., Jiao B. (2014). Decreased expression of microRNA-206 correlates with poor clinical outcome in patients with malignant astrocytomas. *Pathology Oncology Research*.

[14] Tian R., Liu T., Qiao L., Gao M., Li J. (2015). Decreased serum microRNA-206 level predicts unfavorable prognosis in patients with melanoma. *International Journal of Clinical and Experimental Pathology*.

[15] Yang Q., Zhang C., Huang B. (2013). Downregulation of microRNA-206 is a potent prognostic marker for patients with gastric cancer. *European Journal of Gastroenterology & Hepatology*.

[16] Liu X., Zheng W., Zhang X., Dong M., Sun G. (2017). The diagnostic and prognostic value of serum miR-206 in colorectal cancer. *International Journal of Clinical and Experimental Pathology*.

[17] Zhang C., Yao C., Li H., Wang G., He X. (2014). Serum levels of microRNA-133b and microRNA-206 expression predict prognosis in patients with osteosarcoma. *International Journal of Clinical and Experimental Pathology*.

[18] Shi H., Han J., Yue S., Zhang T., Zhu W., Zhang D. (2015). Prognostic significance of combined microRNA-206 and CyclinD2 in gastric cancer patients after curative surgery: a retrospective cohort study. *Biomedicine & Pharmacotherapy*.

[19] Sun P., Sun D., Wang X., Liu T., Ma Z., Duan L. (2015). miR-206 is an independent prognostic factor and inhibits tumor invasion and migration in colorectal cancer. *Cancer Biomarkers*.

[20] Chen A. H., Qin Y. E., Tang W. F., Tao J., Song H. M., Zuo M. (2017). miR-34a and miR-206 act as novel prognostic and therapy biomarkers in cervical cancer. *Cancer Cell International*.

[21] Cui J., Pan Y., Wang J., Liu Y., Wang H., Li H. (2018). MicroRNA-206 suppresses proliferation and predicts poor prognosis of HR-HPV-positive cervical cancer cells by targeting G6PD. *Oncology Letters*.

[22] Hou C. G., Luo X. Y., Li G. (2016). Diagnostic and prognostic value of serum microRNA-206 in patients with gastric cancer. *Cellular Physiology and Biochemistry*.

[23] Ling S., Ruiqin M., Guohong Z., Bing S., Yanshan C. (2015). Decreased microRNA-206 and its function in cervical cancer. *European Journal of Gynaecological Oncology*.

[24] Liu H., Wu H., Qin X. (2019). MicroRNA-206 serves as a tumor suppressor in pediatric acute myeloid leukemia by targeting Cyclin D1. *Pathology, Research and Practice*.

[25] Xue D., Yang Y., Liu Y. (2016). MicroRNA-206 attenuates the growth and angiogenesis in non-small cell lung cancer cells by blocking the 14-3-3*ζ*/STAT3/HIF-1*α*/VEGF signaling. *Oncotarget*.

[26] Guo Z., Jia H., Ge J. (2020). MiR-206 suppresses proliferation and epithelial-mesenchymal transition of renal cell carcinoma by inhibiting CDK6 expression. *Human Cell*.

[27] Chen X. F., Guo J. F., Xu J. F., Yin S. H., Cao W. L. (2019). miRNA-206 inhibits proliferation of renal clear cell carcinoma by targeting ZEB2. *European Review for Medical and Pharmacological Sciences*.

[28] Zhang J., Fa X., Zhang Q. (2019). MicroRNA-206 exerts anti-oncogenic functions in esophageal squamous cell carcinoma by suppressing the c-met/AKT/mTOR pathway. *Molecular Medicine Reports*.

[29] Heinemann F. G., Tolkach Y., Deng M. (2018). Serum miR-122-5p and miR-206 expression: non-invasive prognostic biomarkers for renal cell carcinoma. *Clinical Epigenetics*.

[30] Han Y., Liu M., Wang Z., Huang M., Xu N., Wu L. (2017). Serum microRNAs related with chemoradiotherapy resistance in advanced-stage cervical squamous cell carcinoma. *Translational Oncology*.

[31] Quan Y., Huang X., Quan X. (2018). Expression of miRNA-206 and miRNA-145 in breast cancer and correlation with prognosis. *Oncology Letters*.

[32] Missiaglia E., Shepherd C. J., Patel S. (2010). MicroRNA-206 expression levels correlate with clinical behaviour of rhabdomyosarcomas. *British Journal of Cancer*.

[33] Stang A. (2010). Critical evaluation of the Newcastle-Ottawa scale for the assessment of the quality of nonrandomized studies in meta-analyses. *European Journal of Epidemiology*.

[34] Tierney J. F., Stewart L. A., Ghersi D., Burdett S., Sydes M. R. (2007). Practical methods for incorporating summary time-to-event data into meta-analysis. *Trials*.

[35] Ettinger D. S., Wood D. E., Aisner D. L. (2017). Non-small cell lung cancer, version 5.2017, NCCN clinical practice guidelines in oncology. *Journal of the National Comprehensive Cancer Network*.

[36] Bray F., Ferlay J., Soerjomataram I., Siegel R. L., Torre L. A., Jemal A. (2018). Global cancer statistics 2018: GLOBOCAN estimates of incidence and mortality worldwide for 36 cancers in 185 countries. *CA: a Cancer Journal for Clinicians*.

[37] Ren X. L., He G. Y., Li X. M. (2016). MicroRNA-206 functions as a tumor suppressor in colorectal cancer by targeting FMNL2. *Journal of Cancer Research and Clinical Oncology*.

[38] Liang Z., Bian X., Shim H. (2016). Downregulation of microRNA-206 promotes invasion and angiogenesis of triple negative breast cancer. *Biochemical and Biophysical Research Communications*.

[39] Yang Q., Zhang L., Zhong Y., Lai L., Li X. (2019). miR-206 inhibits cell proliferation, invasion, and migration by down-regulating PTP1B in hepatocellular carcinoma. *Bioscience Reports*.

[40] Pang C., Huang G., Luo K. (2017). miR-206 inhibits the growth of hepatocellular carcinoma cells via targeting CDK9. *Cancer Medicine*.

[41] Chen Z., Gao Y. J., Hou R. Z. (2019). MicroRNA-206 facilitates gastric cancer cell apoptosis and suppresses cisplatin resistance by targeting MAPK2 signaling pathway. *European Review for Medical and Pharmacological Sciences*.

[42] Wang Y., Tian Y. (2018). miR-206 inhibits cell proliferation, migration, and invasion by targeting BAG3 in human cervical cancer. *Oncology Research*.

[43] Dai C., Xie Y., Zhuang X., Yuan Z. (2018). miR-206 inhibits epithelial ovarian cancer cells growth and invasion via blocking c-Met/AKT/mTOR signaling pathway. *Biomedicine & Pharmacotherapy*.

