# Supplementary figures and images for: Prognostic Value and Clinicopathological Features of MicroRNA-206 in Various Cancers: A Meta-Analysis
Source: Biomed Res Int. 2020 Oct 20;2020:2159704. doi: 10.1155/2020/2159704 (PMC7596429; doi:10.1155/2020/2159704)

Study

%

ID

hr (95% CI)

Weight

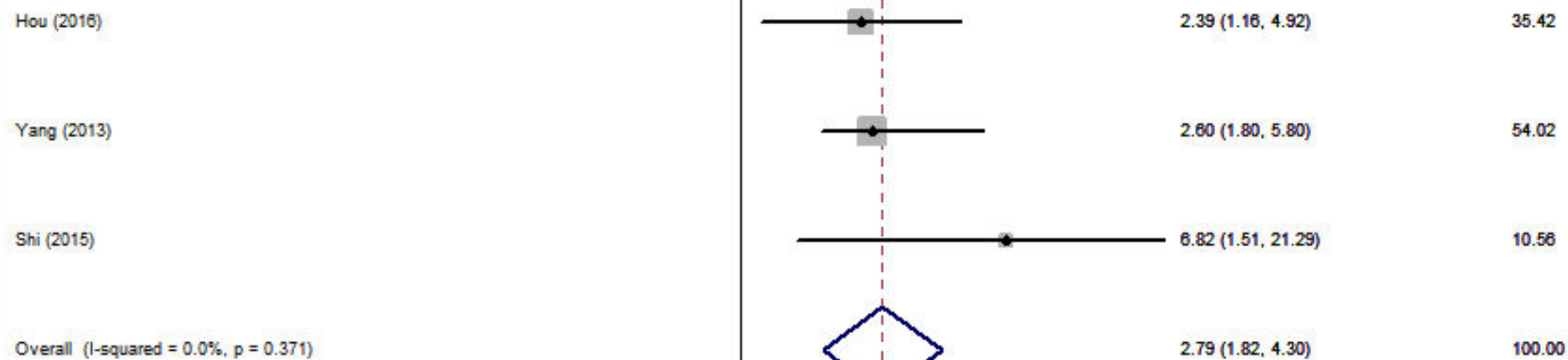

.047

1

21.3

Supplement: Supplementary 1 — Figure S1. Forest plot of the relationship between low miR-206 expression and GC. [file 2159704.f1.pdf]

Study

%

ID

hr (95% CI)

Weight

Liu (2017)

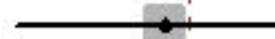

1.75 (1.14, 2.45)

82.38

Sun (2015)

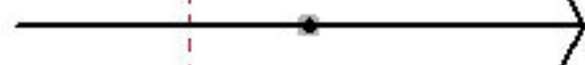

2.68 (1.14, 5.96)

17.62

Overall (I-squared = 0.0%, p = 0.359)

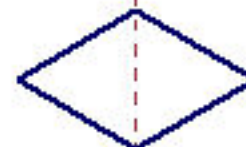

1.89 (1.33, 2.67)

100.00

.168

1

5.96

Supplement: Supplementary 2 — Figure S2. Forest plot of the relationship between low miR-206 expression and CRC. [file 2159704.f2.pdf]

Study

%

ID

HR (95% CI)

Weight

Chen (2017)

1.88 (1.18, 3.01)

41.33

Cui (2018)

1.52 (1.01, 2.28)

54.67

Ling (2014)

6.60 (1.46, 29.70)

3.99

Overall (I-squared = 43.5%, p = 0.170)

1.76 (1.30, 2.38)

100.00

.0337

1

29.7

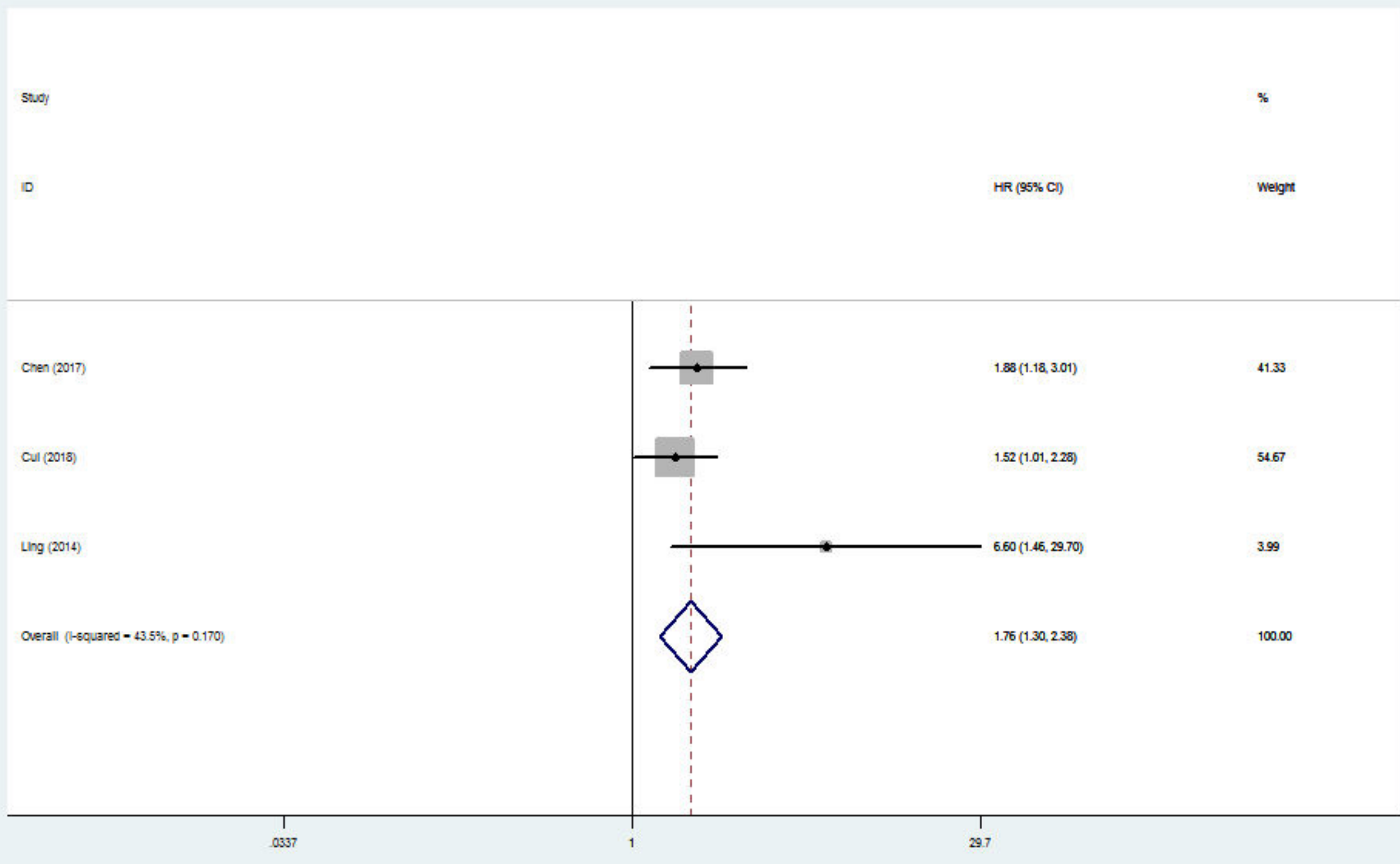

Supplement: Supplementary 3 — Figure S3. Forest plot of the relationship between low miR-206 expression and CC. [file 2159704.f3.pdf]

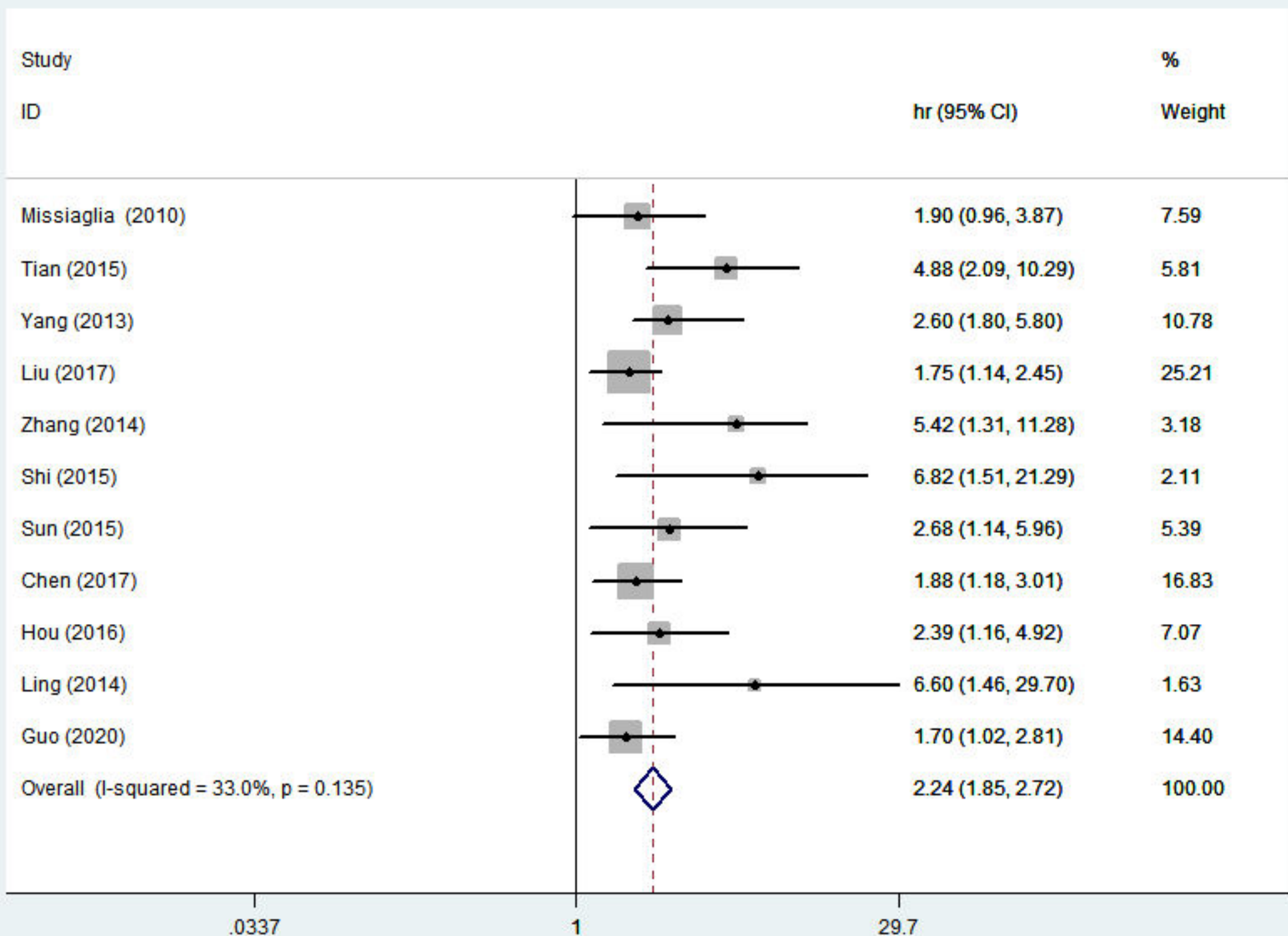

Supplement: Supplementary 4 — Figure S4. Forest plot of subgroup analysis based on multivariate analysis. [file 2159704.f4.pdf]

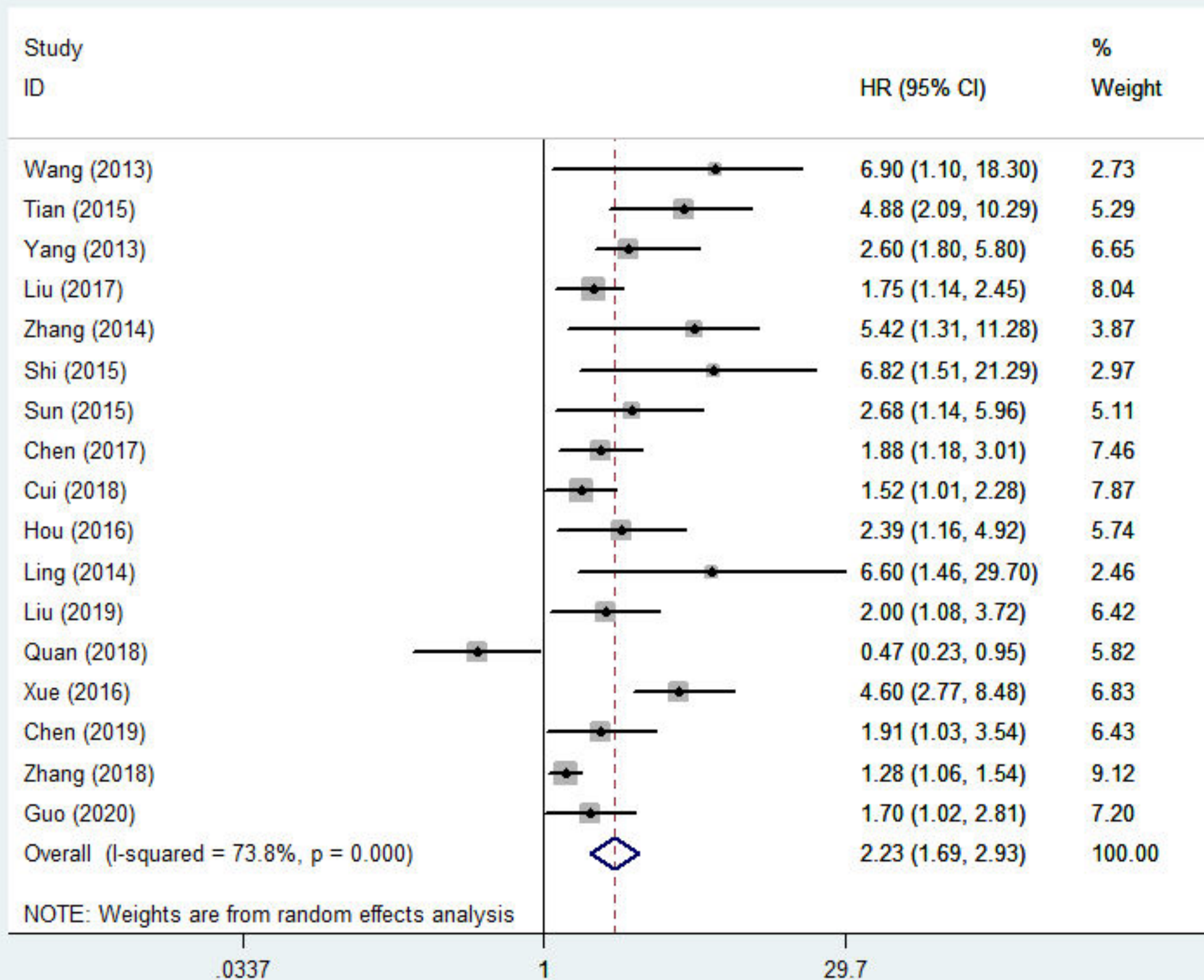

Supplement: Supplementary 5 — Figure S5. Forest plot of subgroup analysis based on Asian. [file 2159704.f5.pdf]

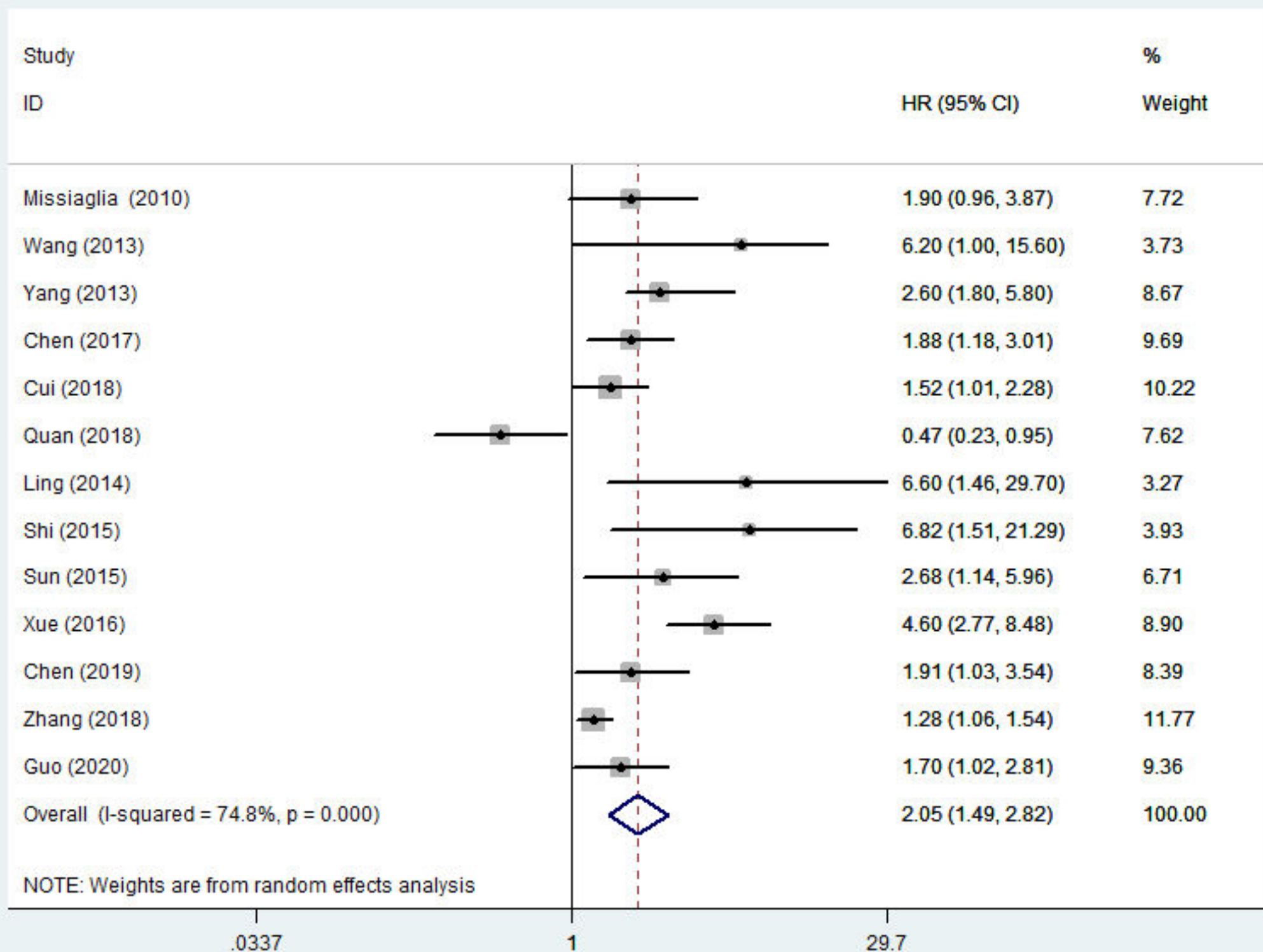

Supplement: Supplementary 6 — Figure S6. Forest plot of subgroup analysis based on tissue. [file 2159704.f6.pdf]

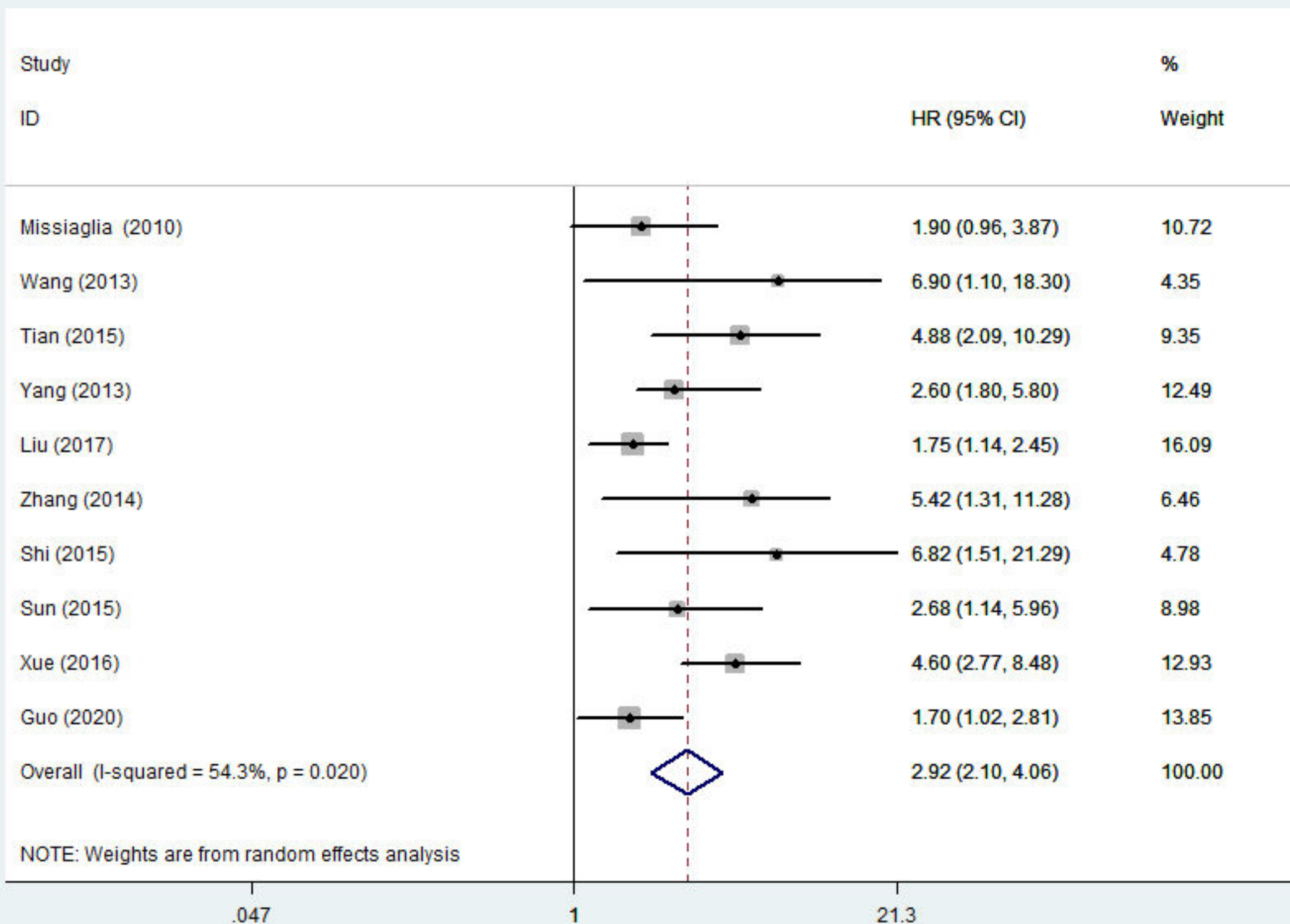

Supplement: Supplementary 7 — Figure S7. Forest plot of subgroup analysis based on data from reported. [file 2159704.f7.pdf]

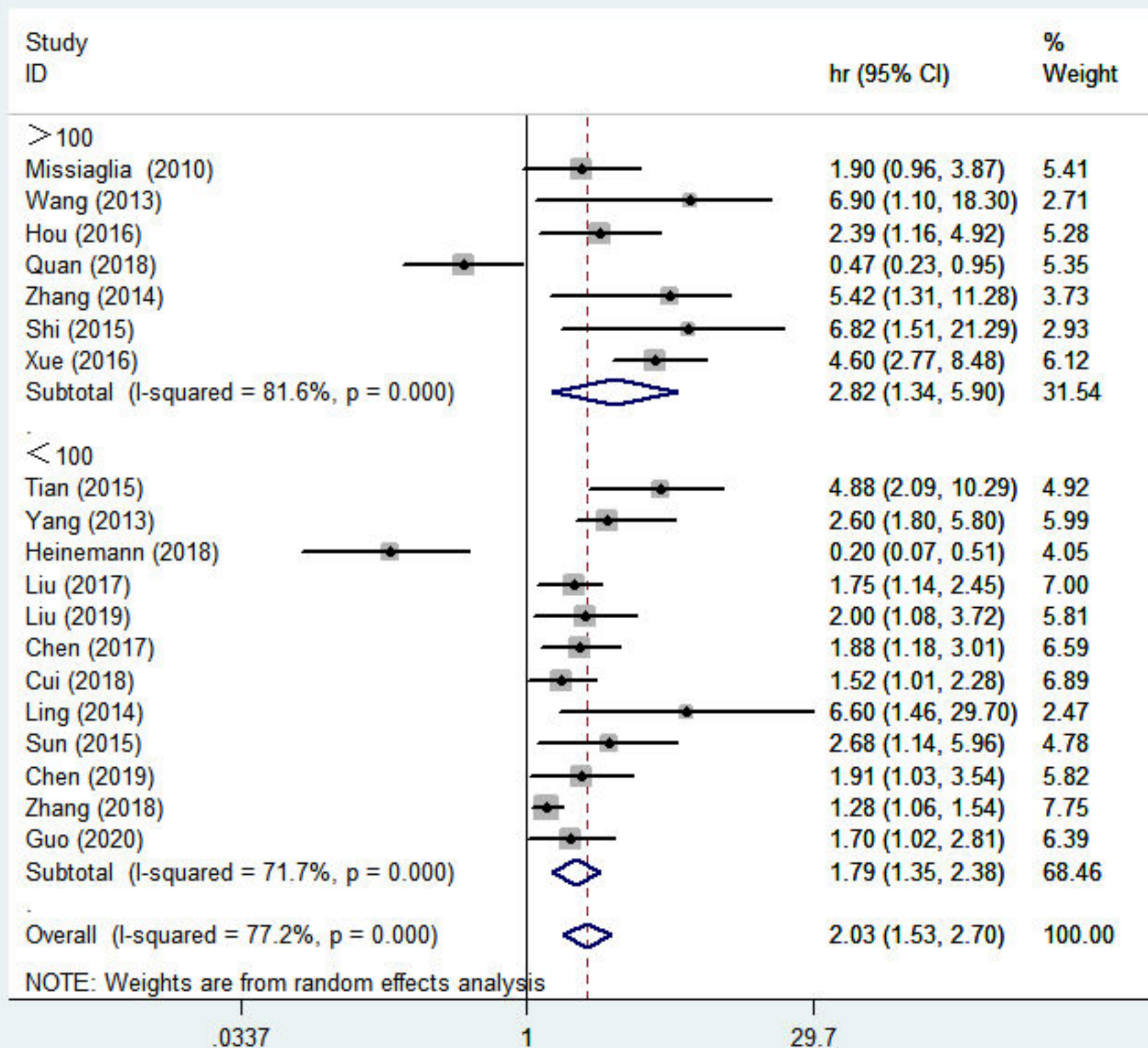

Supplement: Supplementary 8 — Figure S8. Forest plot of subgroup analysis based on sample size. [file 2159704.f8.pdf]
